# Job Loss Due to COVID-19: A Longitudinal Study of Mental Health, Protective and Risk Factors

**DOI:** 10.1177/00332941241248601

**Published:** 2024-04-26

**Authors:** Andrew F. Arena, Daniel Collins, Andrew Mackinnon, Sophia Mobbs, Isobel Lavender, Samuel B. Harvey, Mark Deady

**Affiliations:** Black Dog Institute, Faculty of Medicine and Health, University of New South Wales, Randwick, NSW, Australia

**Keywords:** Job loss, longitudinal, COVID-19, mental health, risk factors, protective factors

## Abstract

The COVID-19 pandemic had a devastating impact on unemployment, which—compounded by the additional stressors associated with the pandemic—had considerable mental health impact. The current study examined the trajectory of mental health amongst those experiencing pandemic-related job loss, alongside the impact of risk and protective factors. Data were obtained from 374 Australian participants who were allocated to a waitlist control arm of a randomised control trial. The outcome variables assessed at baseline and six-month follow-up consisted of depression, anxiety, and suicidality. The assessed risk and protective factors were age, gender, relationship status, education, exercise frequency, COVID-related stress, dispositional resilience, and coping self-efficacy. Re-employment by follow-up was used as a covariate. Overall, there were decreases in depression and anxiety symptoms, and partial evidence of decreased suicidality, demonstrating an apparent capacity for individuals to better cope with their circumstances over time. Demographics and exercise had no effect on changes in mental health. Those with high COVID-related stress, low resilience, and low coping self-efficacy had worse mental health at baseline, although exhibited significantly greater improvements in mental health over time. Obtaining re-employment by follow-up did not predict changes in mental health. The present results offer an optimistic picture of recovery for those experiencing pandemic-related job loss, even for those with the most substantial risk and severity. The likely protective role played by Australian social welfare policies over the course of the study is explored. Stress around one’s broader sociocultural or economic circumstances, perceived resilience, and coping self-efficacy are valuable targets for intervention.

## Introduction

Job loss is commonly ranked among the most stressful or traumatic life events (Cottle, 2001; van Eersel et al., 2019; Walsh, 2007). Research has demonstrated considerably worse mental health outcomes (most commonly increased depression and anxiety) and suicide outcomes for those without employment compared to those with employment, and for those who transition from employment to unemployment (Choi et al., 2022; Gebel & Voßemer, 2014; McKee-Ryan et al., 2005; Milner et al., 2013; Paul & Moser, 2009). Based upon the latent deprivation model, these effects have been largely attributed to the loss of not only fundamental survival needs provided by employment (i.e., financial security), but also the loss of basic psychosocial needs that underpin mental health (i.e., time structure, identity, purpose, activity, and social connection; Creed & Machin, 2002; Creed & Macintyre, 2001; Paul & Batinic, 2010; Paul et al., 2009).

In 2020, within many, but not all countries, the COVID-19 pandemic resulted in an unemployment crisis due to the widespread implementation of social distancing measures that substantially restricted economic activity across the globe (International Labour Organization, 2021). Research confirmed that those experiencing unemployment during the pandemic had poorer mental health outcomes than those who were employed (Lee et al., 2021; Xiong et al., 2020). Australian data revealed up to 8.4 times the odds of experiencing high distress for those unemployed due to COVID-19 compared to those whose work was unaffected, with higher odds for individuals with lower levels of social interaction or financial resources (Griffiths et al., 2021). Furthermore, the experience of unemployment during this time was marked by unique circumstances that added psychological strain; for instance, increased wait times for mental health services, heightened health anxieties, and limited capacity to engage in regular coping activities (e.g., through social interaction; Arena et al., 2022; Bower et al., 2021; Lanz et al., 2023; McDonnell et al., 2022; Tyrer, 2020; Wang et al., 2022). In fact, the unemployment crisis triggered by the pandemic can be conceptualised as an existential crisis for those affected, due to the simultaneous threats to one’s physical safety, one’s basic way of living, and perceptions of the world around them (Blustein et al., 2020). The literature also acknowledges that some of the unique circumstances of the pandemic may have simultaneously protected against negative impacts of unemployment; particularly the normalization of unemployment and increased access to welfare payments seen in many countries during this time (Arena et al., 2022; Grandey et al., 2021). Within the Australian context, the federal government introduced a temporary supplement to unemployment welfare benefits in April 2020 to offset the negative household impacts of pandemic-related lost income (Botha et al., 2022b), which was gradually reduced and ultimately discontinued in March 2021.

Although much is known about the basic prevalence of mental health issues during unemployment, relatively little is known about how mental health develops and changes over time for this population within the unique context of the COVID-19 pandemic. Given that those with longer durations of unemployment commonly have an increased risk of suicide, depression and anxiety outcomes (McKee-Ryan et al., 2005; Milner et al., 2013; Paul & Moser, 2009), and the argument that economic hardship during unemployment leads to cascading disadvantage in other areas (Pharr et al., 2012; Price et al., 1998; Strandh et al., 2014), one may have expected mental health to worsen over time for those experiencing unemployment in the COVID-19 context. Instead, longitudinal research in this field has produced mixed findings internationally (within the USA, UK, South Africa, and Australia), with some support found for deterioration in mental health (Baird et al., 2022; Batterham et al., 2021; Posel et al., 2021), no change in mental health (Baird et al., 2022; Batterham et al., 2021; Botha et al., 2022a; Kessler et al., 2022; Pierce et al., 2020), and improvement in mental health over time (Griffiths et al., 2022; Kessler et al., 2022). Mixed results were found regardless of different designs employed by studies (comparing timepoints before vs. during the pandemic, or during the pandemic only), and most studies used combined depression and anxiety (psychological distress) outcomes, although it is possible that different trajectories could exist for each type of symptom (Batterham et al., 2021). Suicidality has not been examined in the above literature. Furthermore, such divergent results are potentially driven in part by diverse social security policies and experiences of the pandemic in different nations. However, even within the Australian context (Batterham et al., 2021; Botha et al., 2022a; Griffiths et al., 2022), mixed findings suggest that further evaluation is needed to clarify the trajectory of change in mental health for those who lost their job due to the pandemic, in addition to identifying the predictors of such change.

Several risk and protective factors may help to explain individual differences in the impact of COVID-19-related job loss on mental health over time. Certain demographic factors have been shown to play a potential role in minimising the risk of distress during the pandemic for both general and unemployed samples; including older age, male gender, level of education, and being partnered (Baird et al., 2022; Batterham et al., 2021; Botha et al., 2022a; Conversano et al., 2020; Kessler et al., 2022; Pierce et al., 2020; Posel et al., 2021; Proto & Quintana-Domeque, 2021). However, evidence for each of these factors was inconsistent in reaching significance and sometimes emerged in contradictory directions (e.g., both higher and lower levels of education were found to be protective).

Outside of demographic factors, physical exercise is of interest given its prospective relationship with decreased anxiety, depression and suicidality (Brailovskaia et al., 2022; Kandola & Stubbs, 2020; Mammen & Faulkner, 2013). People experiencing unemployment have often been shown to engage in less physical activity compared to those remaining in employment (Biernat & Piątkowska, 2017; Hergenrather et al., 2015; Van Domelen et al., 2011), although such evidence is more mixed in cases of work loss due to the COVID-19 pandemic (Grandey et al., 2021; McDowell et al., 2020), and there is a need for research into the prospective relationship between exercise and mental health within this context. Due to the unique circumstances of the pandemic that added specific stressors for those experiencing job loss (Arena et al., 2022; Blustein et al., 2020; Bower et al., 2021), it is crucial to consider the mental health impacts of specific COVID-19-related stress (Kumar et al., 2022; Lanz et al., 2023). Lastly, dispositional resilience (the general tendency to recover quickly and effectively from stressful events; Smith et al., 2008) and coping self-efficacy (one’s confidence in their ability to engage in effective coping behaviour when facing a stressful event; Chesney et al., 2006) are likely to protect against the negative impacts of sudden job loss. Research has demonstrated their capacity to buffer negative mental health outcomes in general unemployed samples (McGee et al., 2021; Moorhouse & Caltabiano, 2007), although this evidence has tended to be cross-sectional and these variables have not been studied in the context of job loss during the pandemic.

The current exploratory study aimed to clarify the long-term trajectory of mental health outcomes among those who became unemployed due to the COVID-19 pandemic, in addition to the role of potential risk and protective factors (demographic characteristics, exercise frequency, COVID-19-related stress, resilience, and coping self-efficacy). Given the sample, the unprecedented context of the pandemic, and the mixed evidence in the literature, the current study offers novel contributions to the literature on sudden, involuntary job loss, with potential implications for prevention and treatment efforts.

## Methods

### Participants

Participants formed the waitlist control arm of a broader randomized control trial of an Australian digital mental health intervention (*Anchored*; ACTRN12620000178943) were Australian residents aged 18 years or older, recently unemployed due to the COVID-19 pandemic, who owned a smartphone and had good English comprehension. A total of 374 participants responded to baseline questionnaires, with 145 participants having available data on at least one outcome measure at 6-months follow-up (retention rate = 39%). The baseline sample was 67.6% women (32.4% men), 48.9% university educated (35.6% trade certificate or diploma, 15.5% high school or less), 61.8% in a relationship (38.2% single), and had a mean age of 39.33 years (*SD* = 11.90). The most common industries that participants were most recently employed in were accommodation and food services (13.4%), education and training (10.2%), retail (9.1%), healthcare and social assistance (9.1%), and arts and recreation (9.1%). At baseline, 43.3% of respondents had sought professional help for mental health issues within the last month.

## Procedures

All methods were approved by the University of New South Wales (UNSW) Human Research Ethics Committee (HC190914). Participants were recruited into the study through social media advertisements from June to July 2020 (during the peak of the COVID-related unemployment crisis in Australia). All participants were required to provide informed written consent before completing an online baseline assessment. As part of the study, they were then given access to information on mental health and employment support services.

Online follow-up survey links were sent to participants at six months post-baseline, and could be completed within 2 weeks. This follow-up period was chosen based upon the resourcing for the original study, as both feasible and sufficient to capture connections between variables. This period is longer (Batterham et al., 2021; Posel et al., 2021) or as long (Griffiths et al., 2022) as other similarly designed studies in the field. To maximise response rates, participants received up to two reminders to complete the follow-up survey, and were entered into a prize draw to win a $500 gift voucher upon completion.

### Measures

At baseline, participants completed a demographic questionnaire, including information on age, gender, relationship status, highest level of education, and past month professional help-seeking for a mental health problem. All remaining variables were assessed at baseline and follow-up. Exercise frequency was assessed by a single item (“In general, how often do you participate in moderate or intensive physical activity for at least 30 minutes?”), rated on a scale from 1 (*not at all*) to 6 (*every day*). COVID-19-related stress was assessed with a single item (“Which of the following best describes your stress or anxiety levels as they relate to COVID-19?”), rated on a scale from 1 (*none*) to 4 (*severe*).

Dispositional resilience was measured using the 6-item Brief Resilience Scale (BRS; Smith et al., 2008), with higher average total scores capturing a greater self-reported tendency to recover from stress. Within multiple US student and clinical samples (cardiac and chronic pain patients), the scale has demonstrated good internal consistency (Cronbach’s alpha = .80–.91), good test-retest reliability over 1–3 months (ICC = .69–.62), and high convergent validity with related measures (e.g., strong positive correlations with other resilience measures, moderate-strong correlations with positive coping strategies, strong negative correlations with mental health symptoms; Smith et al., 2008).

Coping self-efficacy was measured at baseline using a subset of items from the Perceived Stress Scale (PSS; Cohen et al., 1983). Several studies have interpreted this scale as being comprised of a ‘distress’ factor and a ‘perceived coping ability’ factor, both with sound convergent validity and internal consistency (Hewitt et al., 1992; Martin et al., 1995; Santiago et al., 2019). Thus, the present study retained the items with the greatest content validity for coping self-efficacy (perceived ability to cope with problems, as opposed to distress or perceived controllability of problems). These items asked “In the past month, how often have you…”: (1) “…felt confident in your ability to handle your personal problems?”, (2) “…found that you could not cope with all the things that you had to do?”, and (3) “…felt difficulties were piling up so high that you could not overcome them?”. Responses on these three items therefore reflect coping self-efficacy regarding recent events, with average scores ranging from 0–4, and higher scores denoting greater coping self-efficacy. Strong structural validity was found for this scale (principal components analysis using oblimin rotation confirmed a single factor structure based upon both the scree plot and a single eigenvalue >1, explaining 61% of the variance) in the present sample, alongside good convergent validity with the BRS (*r* = .54, shared variance *r*^2^ = 29%, *p* < .001; suggesting a strong positive relationship, although not so strong as to suggest redundancy in the constructs).

Depression severity over the prior 2 weeks was assessed using the 9-item Patient Health Questionnaire-9 (PHQ-9; Kroenke et al., 2001), which has demonstrated high internal consistency reliability (Cronbach’s alpha = .86–.89), strong criterion validity with diagnostic outcomes, strong sensitivity to change, and strong convergent relationship with other depression and distress scales (Löwe et al., 2004; Martin et al., 2006). Scores range from 0–27, with mild, moderate, moderately severe, and severe symptoms represented by the cut-off points of 5, 10, 15, and 20, respectively (Kroenke et al., 2001).

Anxiety severity over the prior two weeks was assessed with the 7-item General Anxiety Disorder-7 (GAD-7; Spitzer et al., 2006), which has demonstrated strong internal consistency reliability (Cronbach’s alpha = .89–.92), strong criterion validity with diagnostic and functionality outcomes, good structural discrimination from depression scales, and strong convergent relationships with other anxiety and distress scales (Kroenke et al., 2010). Scores range from 0 to 27, with mild, moderate, and severe symptoms represented by the cut-off points of 5, 10, and 15, respectively.

Suicidality over the past month was assessed with the 5-item Suicidal Ideation Attributes Scale (SIDAS; Van Spijker et al., 2014), which is a measure of suicidal ideation based on frequency, controllability, closeness to attempt, distress and interference with daily activities. The scale has demonstrated good internal consistency reliability (Cronbach’s alpha = .86–.91), strong single-factor structural validity, good criterion validity with history of suicidality outcomes, and strong convergent relationships with other suicidality measures (Van Spijker et al., 2014). Scores range from 0–50, with greater suicidality indicated by higher scores, and scores ≥21 reflecting high suicide risk.

### Data Analysis

Baseline predictors of attrition by follow-up were assessed to probe for potential bias in available data. Only one significant difference emerged, with those completing follow-up more likely to be university educated than those not completing follow-up; χ^2^ (1, *N* = 374) = 7.14, *p* = .008. Pearson correlations were calculated for all continuous variables. To firstly test for overall significant changes on all three outcome variables (depression, anxiety, and suicidality) over the follow-up period, repeated measures ANOVAs and ACOVAs were conducted before and after controlling for re-employment by follow-up. For these analyses, SIDAS scores were log (10) transformed due to some departure from normality.

To test for predictors of change in mental health over the follow-up period (i.e., moderation of a within-subjects effect), change scores were first calculated by subtracting baseline outcome scores from follow-up scores (Judd et al., 2001; Magson et al., 2021; Montoya, 2019). No change score variables deviated from normality, and thus no linear transformation of scores was conducted prior to running analyses on these outcomes. Categorical predictors were dummy coded before each baseline predictor variable was entered into separate linear regressions for each outcome, controlling for re-employment by follow-up. To ensure that the effects of the baseline predictors on change in mental health outcomes were not simply reflecting a change in these predictors over time, follow-up levels of the predictor variables were also controlled for in each respective model (excluding demographic predictors). To minimise the impact of missing data, available-case analyses were undertaken using pairwise deletion. Multiple imputation techniques are not advisable in the current context, due to over 40% missing data at follow-up (Jakobsen et al., 2017).

## Results

Descriptive statistics and correlations for all continuous predictors and outcomes are presented in Table 1. Coping self-efficacy tended to have the largest relationships with (positive) mental health outcomes, with a similar pattern of relationships emerging for COVID-related stress and resilience. Age and exercise frequency had small and almost exclusively non-significant relationships with mental health outcomes.

**Table 1.** Descriptive Statistics, Internal Consistency Estimates, and Zero-order Correlations for all Continuous Predictors and Outcomes.

Variables	M (SD)	Range	1	2	3	4	5	6	7	8	9	10	11
1. Age^a^	39.33 (11.90)	18–63	—										
2. Exercise frequency^a^	3.67 (1.43)	1–6	.07	—									
3. COVID-related stress^a^	2.93 (0.71)	1–4	.03	−.02	—								
4. Resilience^a^	2.80 (0.75)	1–5	.02	.16**	−.29**	.84							
5. Coping self-efficacy^a^	1.78 (0.74)	0–4	.09	.19**	−.42**	.54**	.68						
6. Depression T1^a^	13.16 (6.09)	0–27	<.01	−.18**	.51**	−.47**	−.67**	.88					
7. Depression T2^b^	9.97 (6.39)	0–27	−.03	−.09	.28**	−.31**	−.45**	.59**	.90				
8. Anxiety T1^a^	9.81 (5.03)	0–21	−.04	−.07	.45**	−.47**	−.59**	.72**	.40**	.88			
9. Anxiety T2^c^	7.55 (5.08)	0–21	−.11	−.04	.38**	−.38**	−.47**	.54**	.78**	.53**	.90		
10. Suicidality T1^a†^	5.08 (8.74)	0–49	−.05	−.07	.27**	−.39**	−.48**	.54**	.44**	.39**	.39**	.85	
11. Suicidality T2^d†^	4.49 (9.74)	0–48	−.10	−.04	.12	−.38**	−.39**	.44**	.60**	.29**	.51**	.67**	.92

*Note.* *p < .05; **p < .01.

†Descriptives reflect untransformed scores, but correlations were conducted on log(10) transformed scores. Cronbach’s alphas are presented along the diagonal where applicable.

^a^*n* = 374.

^b^*n* = 145.

^c^*n* = 144.

^d^*n* = 142.

At baseline, 22.2% of respondents reported moderately severe and 17.4% severe depression symptoms, 27.2% reported moderate and 20.1% severe anxiety symptoms, and 6.7% reported high risk suicidality. By 6-month follow-up, 13.1% of respondents reported moderately severe and 9% severe depression symptoms, 20.8% reported moderate and 10.4% severe anxiety symptoms, and 6.3% reported high risk suicidality. By follow-up, 68.9% of respondents (*n* = 104/151) were re-employed on either a casual, part-time or full-time basis.

Repeated measures ANOVAs assessed the degree of change in mental health outcomes over time (see Table 2). All outcomes significantly decreased over time prior to controlling for re-employment by follow-up. After controlling for re-employment, reductions in depression and anxiety remained significant, although the reduction in suicidality became non-significant.

**Table 2.** Repeated Measures ANOVAs Testing Changes in Mental Health Outcomes Over 6-Month Follow-Up.

Outcome	Mean Change	SD	Effect size (*d*)	Controlling for re-employment	*F*	*df*	*p*
Depression	−3.37	5.76	0.53	Before	31.11	1, 143	<.001
				After	8.44	1, 142	.004
Anxiety	−2.13	4.94	0.41	Before	17.32	1, 142	<.001
				After	3.98	1, 141	.048
Suicidality	−1.18	6.81	0.18	Before	4.96	1, 140	.028
				After	1.43	1, 139	.234

**Figure 1.**
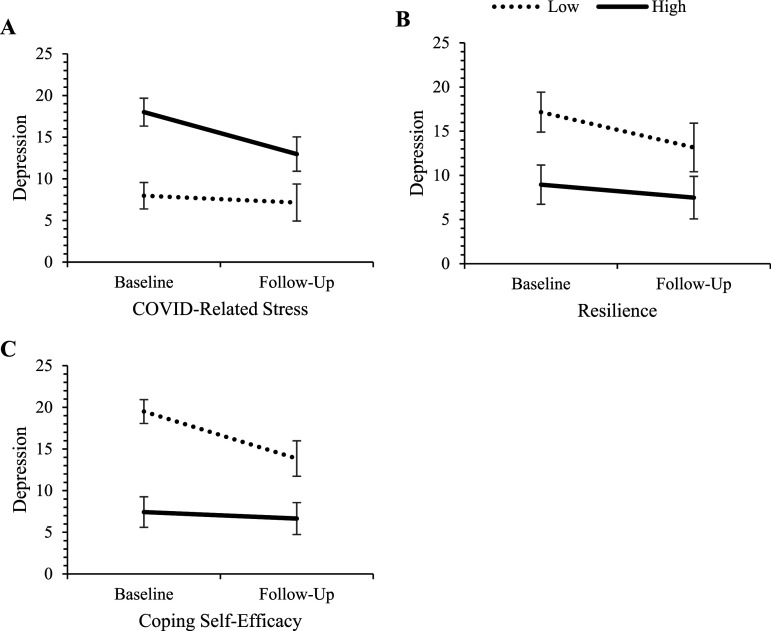
Change in depression for those high and low on COVID-related stress, resilience, and coping self-efficacy. *Note.* ‘High’ and ‘low’ represent values at ±1 SD from the mean of the predictor variables. Error bars represent 95% confidence intervals for depression scores in each cell.

**Figure 2.**
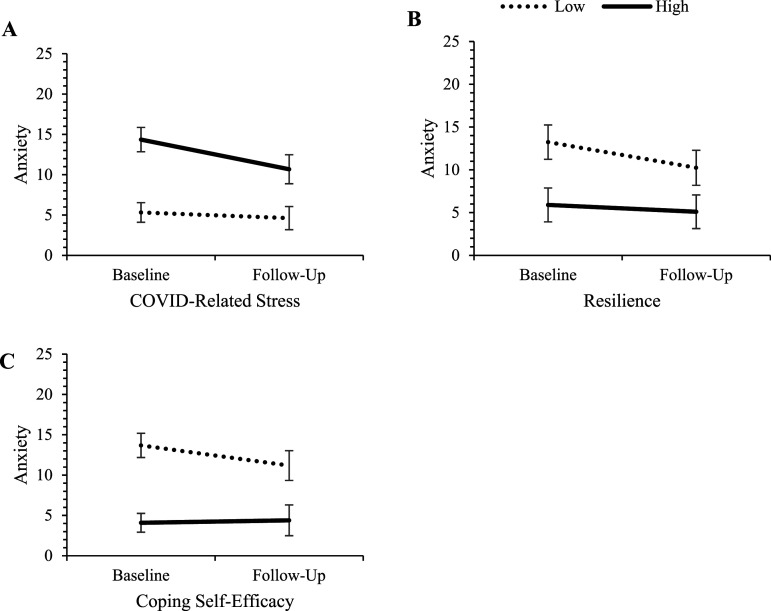
Change in anxiety for those high and low on COVID-related stress, resilience, and coping self-efficacy. *Note.* ‘High’ and ‘low’ represent values at ±1 SD from the mean of the predictor variables. Error bars represent 95% confidence intervals for depression scores in each cell.

**Figure 3.**
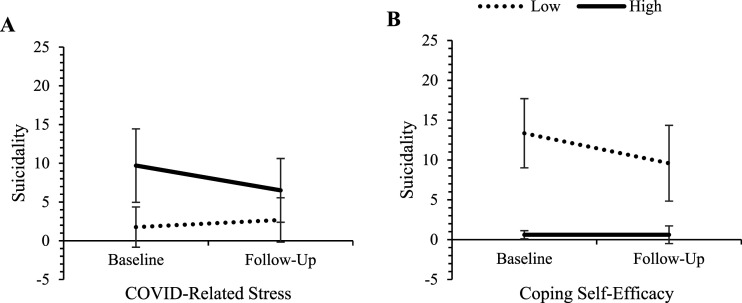
Change in suicidality for those high and low on COVID-related stress and coping self-efficacy. *Note.* ‘High’ and ‘low’ represent values at ±1 SD from the mean of the predictor variables. Error bars represent 95% confidence intervals for depression scores in each cell.

The individual effects of baseline predictors on changes in the three mental health outcomes over the 6-months period (controlling for re-employment by follow-up, and where appropriate, follow-up levels of predictor variables) are presented in Table 3. Controlling for re-employment and follow-up levels of predictor variables had minimal impact on the pattern of predictor effects and did not alter overarching conclusions. No demographic variables, nor exercise frequency, predicted changes in any outcomes. Greater declines in depression and anxiety were found for those who had higher COVID-related stress, lower resilience and lower coping self-efficacy. Greater declines in suicidality were found for those with higher COVID-related stress and lower coping self-efficacy. Further examining the nature of these effects revealed that those who had higher COVID-related stress, lower resilience, and lower coping self-efficacy began the study with poorer mental health but improved to a greater extent. Those with the opposing levels of these predictors began the study with better mental health and remained relatively more stable over the follow-up period. See Figures 1–3 for visual representations of these effects at high and low levels of the predictors (±1SD from the mean).

**Table 3.** Univariable Predictors of Mental Health Change Scores Adjusted for Follow-Up Employment Status.

Predictor	Depression			Anxiety			Suicidality		
	*b* (SE)	*β*	*p*	*b* (SE)	*β*	*p*	*b* (SE)	*β*	*P*
Age	−0.05 (0.04)	−0.11	.196	−0.04 (0.04)	−0.10	.251	−0.01 (0.05)	−0.01	.897
Gender	−1.98 (1.05)	−0.13	.141	−0.15 (0.92)	−0.02	.866	−1.00 (1.26)	−0.07	.428
Relationship status	0.40 (0.99)	0.03	.688	−0.27 (0.85)	−0.03	.756	0.58 (1.17)	0.04	.620
Education	0.19 (0.97)	0.02	.841	0.22 (0.84)	0.02	.796	0.43 (1.15)	0.03	.707
Exercise frequency^a^	0.49 (0.38)	0.12	.191	0.29 (0.33)	0.08	.379	0.43 (0.45)	0.09	.337
COVID-related stress^a^	**−2.62 (0.65)**	**−0.32**	**<.001**	**−1.86 (0.57)**	**−0.27**	**.001**	**−2.33 (0.80)**	**−0.24**	**.004**
Resilience^a^	**3.96 (0.86)**	**0.51**	**<.001**	**2.95 (0.75)**	**0.45**	**<.001**	1.63 (1.08)	0.18	.134
Coping self-efficacy^a^	**4.14 (0.63)**	**0.54**	**<.001**	**3.06 (0.54)**	**0.46**	**<.001**	**2.79 (0.86)**	**0.31**	**.001**

*Note.* Significant effects are bolded for clarity. Gender (0 = woman, 1 = man); Relationship status (0 = single, 1 = in a relationship); Education (0 = university degree, 1 = less than university).

^a^To partial out the effect of change in non-demographic continuous predictors over time, each test additionally controls for follow-up levels of the respective predictor variable.

We also note that the effect of re-employment was non-significant in all models for depression and anxiety outcomes, although was significant in two models for suicidality (testing the effects of gender and COVID-related stress). When entered into the regression models alone, re-employment was not a significant predictor of any outcome: depression *b* (SE) = −0.90 (1.04), *β* = −0.07, *p* = .385; anxiety *b* (SE) = −0.77 (0.89), *β* = −0.07, *p* = .392; suicidality *b* (SE) = −2.26 (1.23), *β* = −0.15, *p* = .067.

## Discussion

The current study investigated the trajectory of depression, anxiety, and suicidality outcomes over 6 months for individuals experiencing job loss due to the COVID-19 pandemic. Depression and anxiety were found to improve over time, and partial evidence was found for improvements in suicidality. This study also uncovered certain risk and protective factors for mental health outcomes, whereby individuals beginning the study with higher COVID-related stress, lower dispositional resilience, and lower coping self-efficacy had greater risk of poor mental health, but also improved to a greater degree over time. Low COVID-related stress, high resilience, and high coping self-efficacy served as protective factors, predicting better mental health outcomes that remained relatively stable over the study period. Demographic factors and exercise frequency had no impact on changes in mental health over time. Several implications stem from these findings that can inform prevention and treatment of mental health issues for individuals experiencing sudden job loss.

The overarching trajectory of mental health and suicidality demonstrates an improvement in outcomes. As suggested by the studied predictors of change, even those who were most vulnerable or unwell at baseline showed sizable capacity for improvement over time, in the absence of any specific therapeutic intervention. This finding may seem unexpected given that longer durations of unemployment are typically associated with greater mental health issues (McKee-Ryan et al., 2005; Milner et al., 2013; Paul & Moser, 2009); however, it aligns with some other research conducted during the pandemic (Griffiths et al., 2022; Kessler et al., 2022). Over the study period, a large proportion of participants became re-employed, yet this could not account for the observed improvements. Re-employment had no noticeable effect on the studied outcomes, which again runs counter to much of the past literature that has tended to find re-employment to have a substantial positive impact on mental health (Gebel & Voßemer, 2014; Paul & Moser, 2009). It is possible that instead, participants became habituated to the unique circumstances of job loss during the pandemic, that are not typical of the general unemployment experience. The circumstances surrounding the job loss were outside of individuals’ control, potentially viewed as temporary, and job loss was much more common during this time, suggesting that job loss was somewhat normalised during the height of the COVID-19 pandemic (Arena et al., 2022). Simultaneously, certain benefits may have emerged for people experiencing job loss at this time, including personal growth and increased compassion (Arena et al., 2022). Evidence regarding work loss during the pandemic also suggests dual pathways leading to higher distress based upon job threat, but also lower distress through increased opportunities for relaxation (Grandey et al., 2021).

Importantly, one factor that potentially contributed to the amelioration of mental health symptoms involves Australian social welfare policies. The study began at the peak of pandemic-related unemployment in Australia, at which point the government had temporarily implemented an additional supplement to unemployment welfare benefits, which remained available during the follow-up period (December 2020–January 2021), although the rate was decreased in September 2020 and again in January 2021 (Botha et al., 2022b). It is known that financial stress accounts for much of the relationship between unemployment and mental health (Backhans & Hemmingsson, 2012; McKee-Ryan et al., 2005), including during the COVID-19 pandemic in Australia (Botha et al., 2022b; Dawel et al., 2020). There is also evidence that the Australian COVID-19 welfare supplement buffered the mental health impacts of financial stress for those experiencing unemployment (Botha et al., 2022b; Bower et al., 2021), which aligns with other evidence regarding the positive mental health impacts of sustained or increased welfare spending during economic recessions (Barr et al., 2015; Deady et al., 2020; World Health Organisation, 2011). Therefore, it is quite plausible that the increased welfare spending of the Australian government greatly buffered the potentially catastrophic mental health impacts of the pandemic for those who lost their job, despite the mass economic disruption caused by the pandemic. After an initial stressful adjustment to their novel circumstances, it is plausible that for participants in the current study receiving the welfare supplement, reduced financial strain may have facilitated recovery—although this argument remains speculative given access to welfare payments was not assessed. If true, this may also partly explain the lack of effect of re-employment, and makes a case for the mental health benefits of increased welfare spending during times of economic instability.

In line with the findings of Griffiths et al. (2022), mental health was worse soon after pandemic-related job loss, which has important implications for prevention and treatment efforts (Carbone, 2020). This is the time that individuals are likely to have the greatest need for mental health support, and potentially when interventions can make the greatest impact—at least in comparable contexts of economic crisis and increased welfare spending. It should be noted that studies finding declines in mental health among those who lost their job due to the pandemic were conducted over the first few months after job loss (Batterham et al., 2021; Posel et al., 2021), rather than over 6 months (Griffiths et al., 2022), implying that recovery may only emerge over longer periods of time. Regardless of whether individuals on average show improvements, there is value in expediting recovery, which could in turn promote earlier re-employment given the bidirectional relationship between unemployment and mental health (Olesen et al., 2013; Stolove et al., 2017). Early intervention and prevention initiatives are therefore crucial, and linking individuals to the right services and resources at the time of job loss ought to be a priority for workplaces. Digital mental health tools are likely to be a valuable solution, in that they have been shown to be effective (Deady et al., 2017), can be immediately accessed at no or low cost, and can avoid stigma-related barriers to help-seeking. However, there are currently no available digital interventions shown to be effective for this population (Arena et al., 2023), and tailoring such interventions to the unique experience of unemployment is critical (Arena et al., 2022).

Low COVID-related stress appeared to be a protective factor for mental health issues experienced over the course of the pandemic. This indicates the importance of considering unique contextualising factors when examining the mental health impacts of job loss, as stress about the pandemic and the added uncertainty of the job market may have compounded the stress of unemployment (Arena et al., 2022). During times of economic crisis, managing stress about the crisis itself appears to be an important target of mental health interventions. COVID-related stress may also partly indicate generally high stress or anxiety, or proneness to stress, as suggested by the high correlations with baseline depression and anxiety; in which case this variable could to some degree be interpreted as an indicator of baseline severity of mental health issues that provides an opportunity for greater attenuation of mental health symptoms.

Dispositional resilience and coping self-efficacy are also valuable targets for protecting against negative mental health outcomes of job loss. People who perceive themselves to be resilient use more effective coping strategies for stress management (Steensma et al., 2007), and during unemployment, dispositional resilience has been found to buffer the negative mental health impact of job searching for extended periods (Moorhouse & Caltabiano, 2007), thus indicating (perhaps unsurprisingly) that resilient traits better equip individuals to cope with the challenges of job loss. Coping self-efficacy as measured in the current study referred to one’s experiences over the prior month, and is therefore more reflective of one’s appraisals of their current situation than the resilience measure, which is more reflective of one’s broader tendency to bounce back from stress. Feeling confident in one’s ability to cope with their present situation is considered prerequisite to enacting known coping strategies (Chesney et al., 2006), meaning that when coping self-efficacy is high, the stressful circumstances of job loss are less likely to result in poor mental health (McGee et al., 2021). The present study builds on past cross-sectional research by demonstrating this protective role longitudinally. Both resilience and coping self-efficacy ought to be considered as focal targets of preventative interventions for those entering the workforce (e.g., Golan et al., 2023) or perhaps early interventions for those experiencing job loss. Indeed, several existing therapeutic techniques could be capitalised on for this purpose, including cognitive behavioural techniques and mindfulness-based approaches for increasing resilience (Kumar et al., 2022), and guided mastery and social modelling of effective coping for coping self-efficacy (McGee et al., 2021). Furthermore, low levels of dispositional resilience and coping self-efficacy could be useful within indicated prevention approaches (Langhammer et al., 2021), as markers of individuals that are more likely to need support in adjusting to sudden job loss.

In the case of demographic risk or protective factors, the present study was unable to support past results or clarify mixed effects found in the literature. Within the present sample, it seems that personality and other psychological individual differences were more important predictors of change in mental health over time. Physical exercise also failed to emerge as a significant protective factor. Although this runs counter to certain expectations based on the literature (Mammen & Faulkner, 2013), some studies have shown that non-exercise recreational or relaxation activities can have a stronger relationship with improved mental health during unemployment (Goodman et al., 2016; Grandey et al., 2021). Thus, it is possible that leisure-based activities that are self-focused, social, or relaxing, rather than solely physical, ought to form part of the recommendations for people experiencing job loss.

### Limitations and Future Directions

Although a key strength of the current study is its long-term follow-up of individuals who lost their job due to the pandemic, the lack of pre-job loss baseline data limits the inferences that can be drawn. The available data cannot determine whether the observed improvements in mental health are indicative of a return to pre-job loss levels, or whether mental health remains lower or higher than before the pandemic. This is a key advantage of existing nationally representative panel surveys, although such approaches are often subject to their own limitations, for example, changes in methodology between assessments (Botha et al., 2022a; Pierce et al., 2020). Participant attrition was also an issue within the study, which may have biased the results to some extent. We note that there were minimal observable baseline differences between participants available and unavailable at follow-up, yet the observed difference in education cannot be adjusted for within the current study’s design, and so constitutes a limitation. The present study cannot rule out the influence of repeated measurements on the observed improvements over time, in that participants may have expected improvements and responded accordingly. Equally, these individuals had registered to take part in an evaluation of a mental health/wellbeing app, and thus may not be representative of all those unemployed at this time but, conversely, are likely to be a more mentally unwell group and thus findings might best be viewed in this context.

The current study did not assess financial stress or whether participants were receiving welfare benefits, which allows for only speculative conclusions about role of these factors in driving improvements over time. Furthermore, Australia encountered lower rates of COVID-19 infection relative to many other countries, but relatively harsher lockdowns, which also varied by region, and so care must be taken when extrapolating conclusions to other contexts. Lastly, the current sample is highly heterogenous in terms of occupational background, and so it is possible that effects may differ within specific industries that this study is not well-suited to explore.

## Conclusions

The present study found that over 6 months, the mental health of people who lost their job due to the COVID-19 pandemic generally improved. The greatest need for mental health support was seen in the early stages of job loss, particularly for those with higher COVID-related stress, lower dispositional resilience, and lower coping self-efficacy. It is recommended that clinicians, employment services providers, and governments aiming to protect against the negative mental health impacts of sudden, uncontrollable job loss, focus on managing stress around one’s broader sociocultural or economic circumstances, and building positive self-evaluations around one’s resilience and ability to cope.

## Data Availability

The raw data are not publicly available in accordance with data security measures approved by the relevant ethics committee. Further information on the data can be made available upon reasonable request from the corresponding author.*
